# Use of Electronic Death Certificates for Influenza Death Surveillance^1^

**DOI:** 10.3201/eid2001.130471

**Published:** 2014-01

**Authors:** Elizabeth A. Bancroft, Sun Lee

**Affiliations:** Los Angeles County Department of Public Health, Los Angeles, California, USA

**Keywords:** influenza, pandemic, mortality, surveillance, death certificates, Los Angeles, deaths, viruses

## Abstract

Surveillance for influenza deaths has been used to gauge the severity of influenza seasons. Traditional surveillance, which relies on medical records review and laboratory testing, might not be sustainable during a pandemic. We examined whether electronic death certificates might provide a surveillance alternative. We compared information retrieved from electronic death certificates that listed influenza (or a synonym) with information retrieved from medical charts on which influenza deaths were reported by traditional means in Los Angeles County, California, USA, during the 2009 influenza A(H1N1) pandemic and 2 subsequent influenza seasons. Electronic death certificate surveillance provided timely information, matched the demographics and epidemiologic curve of that obtained from traditional influenza-related death surveillance, and had a moderately positive predictive value. However, risk factors were underreported on death certificates. Because surveillance by electronic death certificates does not require obtaining and reviewing medical records, it requires fewer resources and is less burdensome on public health staff.

## Abstract 2

These certificates might be useful during a pandemic, when resources for traditional surveillance are limited.

Each year in the United States, more deaths are estimated to be caused by influenza than by AIDS (*1*,*2*). Influenza viruses commonly mutate, and concern that a new influenza pandemic will arise is always present. Hence, extensive clinical, syndromic, and virologic surveillance for influenza is conducted in the United States and worldwide. For determining the severity of each influenza season, recording the number of deaths from influenza has long been part of the national system. Although in the United States, most estimates of influenza deaths use a complex algorithm involving data from death certificates and virologic surveillance, in select situations, case reports of individual deaths are used (*3*,*4*). For example, since 2004, influenza-related deaths among children have been nationally reportable, and during the 2009 influenza A(H1N1) pandemic, laboratory-confirmed influenza-related deaths among persons of all ages were reported by state health departments to the Centers for Disease Control and Prevention (*5*,*6*).

Individual case reports (ICRs) of laboratory-confirmed influenza-related deaths provide useful information about the strain of influenza that caused the death, the demographic characteristics of the persons who died, and traditional and novel risk factors for death (*7*–*9*). Deaths are initially reported to health departments by hospitals, physicians, and medical examiners. Health departments collect medical records, laboratory results, specimens for confirmation at public health laboratories, and occasionally interviews of health care providers to determine whether the initial case report meets the definition of a laboratory-confirmed influenza-related death (*5*). 

However, collecting and reviewing detailed medical records and laboratory confirmation reports can be time-consuming and labor-intensive. During pandemics, the infrastructure and resources needed to perform public health surveillance of individual influenza deaths can become limited right when the demand for knowledge about disease trends increases. Resources for performing a full, or even limited, investigation of individual influenza-associated deaths might not be available (*10*). Therefore, during pandemics, automated surveillance systems might prove useful for influenza death surveillance.

To evaluate usefulness of an automated influenza death reporting system during and after the 2009 influenza A(H1N1) pandemic, we investigated all death certificates in Los Angeles County, California, USA, on which influenza was listed as a direct or indirect cause of death from August 2009 through April 2012. We compared the sensitivity, positive predictive value, and timeliness of an electronic death reporting system (EDRS) with that of traditional influenza death surveillance based on ICRs.

## Methods

### Case Finding and Case Definitions

#### ICRs 

Since April 2009, all influenza deaths have been reportable to the Los Angeles County Department of Public Health. For this study, initial ICRs were received by fax and by electronic web reporting from infection prevention personnel at hospitals, private medical practices, the coroner’s office, and other jurisdictions. For each initial ICR, Department of Public Health staff obtained medical records and laboratory results; from these original medical records, trained staff at the Los Angeles County Department of Public Health extracted demographic and risk factor information by using a standard form. Information extracted included the presence of congenital and/or neurologic conditions, immunosuppressive conditions, diabetes, cardiovascular or pulmonary conditions, renal dysfunction, patient’s height and weight, and other information. Obesity was defined as body mass index >30 for adults or >95th percentile for those <19 years of age. A death reported on an ICR was defined as death of a person 1) who resided in Los Angeles County (except for the cities of Long Beach and Pasadena, which maintain their own health departments); 2) whose influenza infection was considered in whole, or in part, to have contributed to the death; and 3) who had a documented positive influenza test result. Laboratory tests included virus culture, PCR, direct or indirect fluorescent antibody test, or a rapid test.

#### EDRS

Since October 2007, all deaths that occur in Los Angeles County (except those that occur in Long Beach and Pasadena) are recorded by the electronic filing of death certificates into the EDRS at Los Angeles County Department of Public Health. As part of the 122 Cities Mortality Reporting System, each week an algorithm is used to search the EDRS at the Los Angeles County Department of Public Health for death certificates on which influenza was listed as the underlying or contributing cause of death (*11*). Because death certificates are searched at the time of initial filing at Department of Public Health, at that time they have not yet had been coded by the International Classification of Diseases, Tenth Revision, for causes of death. Therefore, specific text strings representing influenza or its synonyms (e.g., flu, swine, H1N1, H1, HINI, N1H1) are used to identify influenza in the cause of death or other significant conditions sections by using SAS software version 9.2 (SAS Institute, Cary, NC, USA). As indicated in the 122 Cities Mortality Reporting System guidelines for reporting influenza, any certificates that mention death from *Haemophilus influenzae* and/or parainfluenza virus are excluded. 

For this study, trained staff at the Los Angeles County Department of Public Health used a standard form to extract information about demographics and risk factors from the death certificates. Information extracted included the presence of congenital and/or neurologic conditions, immunosuppressive conditions, diabetes, cardiovascular or pulmonary conditions, renal dysfunction, and other conditions. A case-patient was recorded as obese if the word “obese” or “obesity” was listed on the death certificate. The EDRS defined an influenza death as death of a person who 1) died in Los Angeles Country, regardless of his/her residence, and 2) had influenza (or a synonym listed above) recorded on the death certificate in the week during which the death certificate was originally filed. Medical records and laboratory results were also obtained for each influenza death reported by the EDRS, data were abstracted by using the same form used for deaths reported on ICRs, and cases were reviewed by trained staff at the Los Angeles County Department of Public Health to determine whether the influenza death reported in the EDRS met the case definition for an influenza death reported on ICRs.

### Analysis

The pandemic period was defined as August 30, 2009, through April 30, 2010. To determine whether the 2 surveillance systems provided similar descriptions of the demographics, underlying conditions, and trends of influenza deaths, we used *t*-tests, χ^2^ tests, and Fisher exact tests, as appropriate, to compare their results. 

We calculated the timeliness of each surveillance system for deaths that occurred during the pandemic period. For cases reported by ICR, lag time was defined as the number of days that elapsed from the date of death until the date the death was confirmed by medical and laboratory record review as being influenza related. Lag time was calculated only if the case had been reported at the time of or after death (cases that had initially been reported before death, as part of surveillance for influenza-related intensive care unit admissions, were removed from this analysis). For cases reported by the EDRS, lag time was defined as the number of days that elapsed between the date of death and the date that the death was identified.

For the pandemic period and for the subsequent 2 influenza seasons combined (August 2010–July 2012), we calculated the sensitivity and positive predictive value for the EDRS and used the ICR system as the standard. Data were stored in Microsoft Access (Redmond, WA, USA) databases and analyzed by using SAS software version 9.3 (SAS Institute).

Cases were identified as part of routine public health surveillance for influenza-related deaths. As such, no explicit ethical approval was necessary or sought for this study. A high standard of patient confidentiality was maintained.

## Results

During 2009–2010, a total of 105 influenza-related deaths were reported by ICRs and 85 by the EDRS; reported patient demographic characteristics were similar (Table 1), specifically, percentages of female patients (53% and 54%), percentages of Hispanic patients (51% and 56%), and median patient ages (47 and 49 years), respectively. However, risk factors for influenza death were recorded on only 53 (62%) of the 85 electronic death certificates but on 102 (97%) of the 105 ICRs (Table 2).

**Table 1:** Demographic variables associated with influenza-related deaths obtained by 2 surveillance systems, Los Angeles County, California, USA, 2009–2010*

Demographic	Surveillance system	p value

EDRS, n = 85	ICR, n = 105
Age, y			>0.8
Mean	45.4	45.1	
Median	49	47	
Range	0–90	0–94	
Sex, no. (%)			>0.5
M	37 (44)	50 (48)	
F	48 (56)	55 (52)	
Race, no. (%)†			>0.3
Asian	6 (7)	6 (6)	
Black	5 (6)	9 (9)	
Latino	48 (56)	54 (51)	
White	25 (29)	28 (27)	
Other	1 (1)	2 (2)	
Unknown	0	6 (6)	

*EDRS, electronic death reporting system; ICR, individual case report. †Percentages may add to >100% because of rounding.

**Table 2:** Comparison of underlying variables associated with influenza-related deaths obtained by 2 surveillance systems, Los Angeles County, California, USA, 2009–2010*

Condition†	Surveillance method, no. (%)

EDRS	ICR
Any	53 (62)	102 (97)
Developmental disability	3 (4)	13 (12)
Pregnancy	1 (1)	3 (3)
Obesity	2 (2)	52 (50)

*EDRS, electronic death reporting system; ICR, individual case report. †p<0.002.

**Figure 1:**
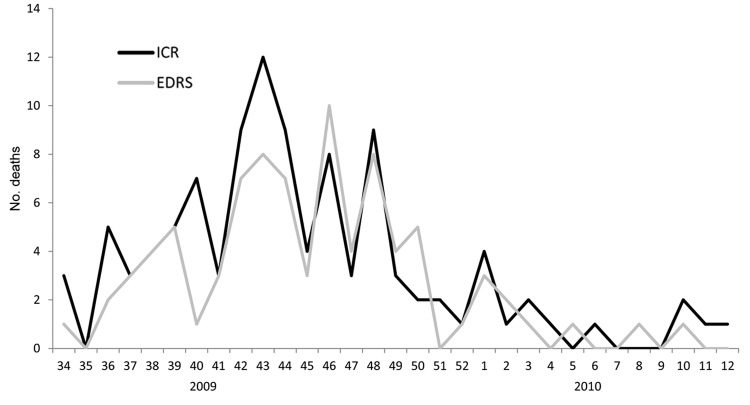
Influenza-related deaths by MMWR (Morbidity and Mortality Weekly Report) week of death reported by 2 surveillance systems, Los Angeles County, California, USA, 2009–2010.

The dates of death reported by each of the 2 surveillance systems were similar (Figure 1). Each system showed peak activity during surveillance weeks 43–48 in 2009 (corresponding to October 18, 2009–December 5, 2009) and low activity during 2010.

The lag times for confirming (ICR) or identifying (EDRS) influenza-related deaths were also similar for the 2 systems (Table 3). After 56 of the 105 ICR deaths with negative lag times were excluded, the mean and median reporting lag times for deaths reported by ICR (n = 49) were 24 and 4 days, respectively (range 0–325 days, interquartile range 1–16 days), and the mean and median reporting lag time for deaths reported by the EDRS (n = 85) were 16 and 11 days, respectively (range 3–86 days, interquartile range 9–16 days). Of the deaths reported by ICR, 5 were reported >50 days after death because of extensive testing at the coroner’s office, which increased the mean lag time for deaths reported by ICR, but the difference between the reported mean lag times was not statistically significant.

**Table 3:** Comparison of lag times associated with reporting influenza-related deaths obtained by 2 surveillance systems, Los Angeles County, California, USA, 2009–2010*

Reporting delay, d†	Surveillance method

EDRS, n = 85	ICR, n = 49
Mean	16	24
Median	11	4
Range	3–86	0–325

*EDRS, electronic death reporting system; ICR, individual case report. †p>0.20.

**Figure 2:**
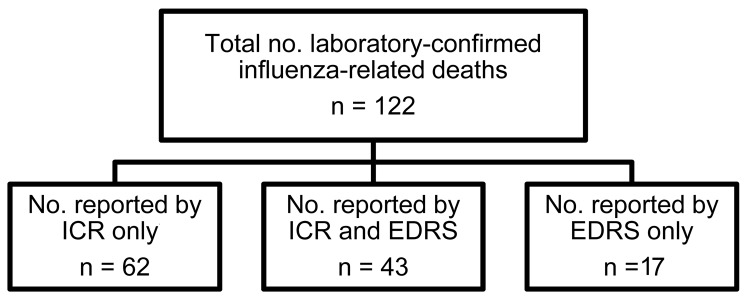
Report source of laboratory-confirmed influenza-related deaths in Los Angeles County, California, USA, April 2009–April 2010. ICR, individual case report; EDRS, electronic death reporting system.

Of the 85 deaths identified by the EDRS during the pandemic period, 60 met the ICR definition for influenza-related death; positive predictive value was 71%. Of the 25 deaths identified by the EDRS that did not meet the case definition, 14 did not have a positive influenza laboratory test result, 10 patients did not live in Los Angeles County although they died in Los Angeles County, and 1 patient was mistakenly selected by the automated algorithm as having influenza although the causative organism listed was *H. influenzae*. A total of 43 of the 105 of the deaths identified by ICR were reported by the EDRS; sensitivity was 41% (Figure 2). Of note, an additional 17 deaths identified by the EDRS met the ICR case definition after medical record review, but these had not been reported to the Los Angeles County Department of Public Health through normal reporting mechanisms.

**Figure 3:**
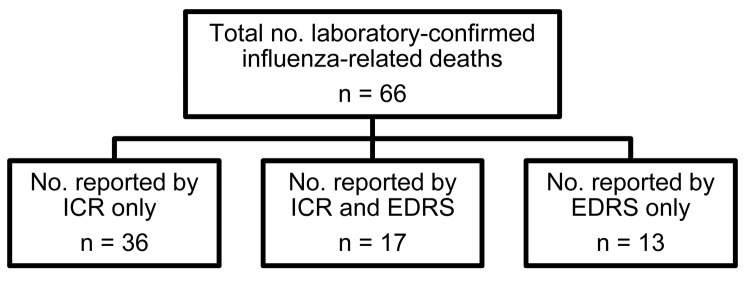
Report source of laboratory-confirmed influenza-related deaths in Los Angeles County, California, USA, August 2010–April 2012. ICR, individual case report; EDRS, electronic death reporting system.

During the postpandemic period (August 2010–July 2012), totals of 53 and 35 influenza-related deaths were reported by ICRs and the EDRS, respectively. Of the 35 deaths reported by the EDRS, 30 were verified as laboratory-confirmed influenza-related deaths of Los Angeles County residents. During the postpandemic period, the sensitivity of the EDRS compared with that of ICRs was 45% and the positive predictive value was 86% (Figure 3).

## Discussion

This study demonstrated that surveillance that used automated text searches of electronic death certificates was timely, matched the demographics and the epidemiologic curve of traditional influenza-related death surveillance in Los Angeles County, and had a moderate positive predictive value. Because no medical records needed to be obtained or reviewed, surveillance by the EDRS required fewer resources and was less burdensome on public health staff and hospitals.

 The major limitation of using death certificates as a primary surveillance tool is that relatively fewer risk factors (including obesity or neurodevelopmental diagnoses, which were newly identified risk factors for severe influenza during the 2009–2010 influenza A[H1N1] pandemic) were listed on the death certificates than on ICRs (*8*,*12*). For example, although 50% of the confirmed deaths reported by ICRs were actually in obese persons, the death certificates rarely listed obesity as being a cause of death or as another significant condition. For identifying obesity as a risk factor for severe complications of influenza A(H1N1) virus infection, medical record review and additional efforts to contact hospitals were needed to obtain the height and weight of the patients. Furthermore, the sensitivity of the EDRS was <50% compared with that of traditional reporting, suggesting that many doctors do not consider influenza as a significant condition to list on death certificates or are not aware of a positive laboratory result when the death certificate is signed.

The number of influenza deaths reported to a health department by any means (death certificates or ICRs) almost certainly underestimates the true number of influenza deaths in a population (*13*). It reflects only those patients who were tested, had adequate specimens for testing, had sensitive tests performed, and were reported to the health department. A recent study estimated that the total number of deaths from pandemic influenza in the Americas was 2–4 times that of reported laboratory-confirmed cases (*14*). Consequently, estimates of the total number of influenza-related deaths in the United States are routinely made through a statistical algorithm based, in part, on standardized International Classification of Disease, Tenth Revision, codes indicated on death certificates, including codes for influenza, pneumonia, respiratory disease, or cardiac disease (*3*,*4*). Because death certificates must be standardized and coded, years can pass between the end of an influenza season and the final estimate of influenza-related deaths that occurred during that season. Therefore, contemporary data, even if underestimated, from ICRs or from death certificates that have not yet been standardized can be useful for local influenza control policies by estimating relative virulence of a given influenza season and identifying risk factors and demographic groups at highest risk for death.

This study has several limitations: it was performed in only 1 jurisdiction, death certificate coding practices might differ in other jurisdictions, and the overall sample size was small. However, Los Angeles County has a diverse population of 9.8 million and multiple reporting sources (≈100 hospitals and ≈25,000 physicians), so the base of reporting was broad and diverse. Because the 122 Cities Mortality Reporting System/EDRS algorithm for identifying influenza and pneumonia on death certificates is processed only 1 time each week, there is a built-in time lag between when the death certificate is filed and when a death is identified in EDRS. Furthermore, because the system looks only at the first time a death certificate is filed (often before cause of death has been determined), it might miss death certificates that are later amended to cite influenza as a cause of death. In fact, of the 105 laboratory-confirmed ICR deaths, 16 were reported by the Office of the Coroner in Los Angeles County. For all 16 patients, death certificates were initially filed with cause of death deferred. Thus, these deaths were not detected by our weekly EDRS search algorithm, although for 13 (81%) of the 16, influenza was ultimately listed on the amended death certificates. To enhance the timeliness and sensitivity of EDRS surveillance, the database should be analyzed more often than 1 time each week and should include amended death certificates. Furthermore, text strings in death certificates should be manually evaluated at regular intervals to ensure that all appropriate terms are included when searching for possible influenza-related deaths.

In summary, as an increasing number of jurisdictions have or plan to use EDRSs (*15*), public health officials might find an EDRS useful for the surveillance of influenza or other emerging diseases. Surveillance that used electronic death certificates and a text-based search algorithm for influenza was able to accurately describe the population affected by influenza during the influenza A(H1N1) 2009 pandemic in a timely and efficient manner. During a pandemic, when surveillance resources can be overwhelmed, use of electronic death certificates to identify and analyze influenza deaths might be a reasonable option. At a minimum, investigation of influenza deaths reported by death certificates might identify additional confirmed influenza-related deaths not reported through traditional mechanisms.
